# Provider perceptions of barriers and facilitators to care in eating disorder treatment for transgender and gender diverse patients: a qualitative study

**DOI:** 10.1186/s40337-023-00760-9

**Published:** 2023-03-08

**Authors:** Katarina A. Ferrucci, Emily McPhillips, Kate L. Lapane, Bill M. Jesdale, Catherine E. Dubé

**Affiliations:** 1 Clinical and Population Health Research Program, Morningside Graduate School of Biomedical Sciences, University of Massachusetts Chan Medical School, 55 Lake Ave North, Worcester, MA 01655 USA; 2 Department of Population and Quantitative Health Sciences, University of Massachusetts Chan Medical School, 55 Lake Ave North, Worcester, MA 01655 USA

**Keywords:** Transgender, Gender diverse, Eating disorders, Treatment, Provider

## Abstract

**Background:**

The prevalence of eating disorders is higher in transgender and non-binary compared to cisgender people. Gender diverse people who seek eating disorder treatment often report struggling to find affirming and inclusive treatment from healthcare clinicians. We sought to understand eating disorder care clinicians’ perceptions of facilitators of and barriers to effective eating disorder treatment for transgender and gender diverse patients.

**Methods:**

In 2022, nineteen US-based licensed mental health clinicians who specialized in eating disorder treatment participated in semi-structured interviews. We used inductive thematic analysis to identify themes around perceptions and knowledge of facilitators and barriers to care for transgender and gender diverse patients diagnosed with eating disorders.

**Results:**

Two broad themes were identified: (1) factors affecting access to care; and (2) factors affecting care while in treatment. Within the first theme, the following subthemes were found: stigmatization, family support, financial factors, gendered clinics, scarcity of gender-competent care, and religious communities. Within the second theme, prominent subthemes included discrimination and microaggressions, provider lived experience and education, other patients and parents, institutions of higher education, family-centered care, gendered-centered care, and traditional therapeutic techniques.

**Conclusion:**

Many barriers and facilitators have potential to be improved upon, especially those caused by clinicians’ lack of knowledge or attitudes towards gender minority patients in treatment. Future research is needed to identify how provider-driven barriers manifest and how they can be improved upon to better patient care experiences.

**Supplementary Information:**

The online version contains supplementary material available at 10.1186/s40337-023-00760-9.

## Abstract (summary)

Eating disorders are more common among transgender and non-binary compared to cisgender people. Despite this, gender diverse people who seek eating disorder treatment often report struggling to find affirming and inclusive care. We interviewed nineteen US-based licensed mental health clinicians who specialized in eating disorder treatment to learn perceptions and knowledge of facilitators and barriers to care for transgender and gender diverse patients diagnosed with eating disorders. Factors affecting access to care included stigmatization, family support, financial factors, gendered clinics, scarcity of gender-competent care, and religious communities. Factors affecting care while in treatment included discrimination and microaggressions, provider lived experience and education, other patients and parents, institutions of higher education, family-centered care, gendered-centered care, and traditional therapeutic techniques. This research identified barriers and facilitators with the potential for improvement, especially those caused by clinician’s lack of knowledge or attitudes towards gender minority patients in treatment.

**Supplementary Information:**

The online version contains supplementary material available at 10.1186/s40337-023-00760-9.

## Background

In the United States, 14.4 million people have had an eating disorder (ED) in their lifetime [1]. Self-reported history of EDs has been found to occur among 7.4–17.6% of transgender individuals generally [2–4] and among 4.32% of transgender individuals who have received gender affirming medical intervention [5]. Transgender and gender diverse people (gender minorities) are those whose gender identity differs from the gender they were assigned at birth. Some gender diverse people identify as neither a man nor a woman, or as a combination of male and female, and may use one or several terms (i.e., non-binary, gender fluid, demi-girl, demi-boy) to describe their gender identity [6]. The prevalence of EDs among transgender people is greater than among people whose gender identity aligns with gender assigned at birth (cisgender) [2].

Gender minority individuals often struggle to find healthcare that is both affirming and inclusive [7–10]. Some delay healthcare visits due to fear of discrimination. Depression and suicide attempts are much higher among those who delay healthcare visits out of fear of discrimination relative to those who do not [7]. Those seeking mental health care might be plagued with added stressors. A study using the 2015 United States Transgender Survey reported that nearly 25% of gender minority respondents had to educate their provider on gender identity [11]. Further, 18.8% of transgender women, 21.1% of transgender men, 72.5% of nonbinary, assigned female at birth, and 65.6% of nonbinary, assigned male at birth did not feel respected by their provider after identifying themselves as transgender [11]. Despite these challenges, more than half develop relationships with health care clinicians with whom they are very satisfied [12].

Qualitative studies reporting the narratives of gender minority patients seeking and receiving treatment for an ED are consistent with reports based on overall engagement with the health care system. Many describe instances of having received discriminatory comments and microaggressions from clinicians and other members of the care team. Patients emphasize experiencing fear and anxiety due to uncertainty about how inclusive a provider may be especially when attending appointments with specialists (e.g., nutritionist, internists, dentists) whom they were referred to by their primary ED care provider [3, 13, 14]. In response to the need to improve care provided to gender minority patients, the American Psychological Association developed provider standards of care for gender competent treatment [15]. Yet, analyses of patient narratives derived from two different projects led by FEDUp (Fighting Eating Disorders in Underrepresented Populations) reveal these standards of care may not have diffused into clinical practice [3, 13]. Transgender patients seeking and receiving eating disorder treatment felt that the clinicians with whom they engaged were generally unfamiliar with specific needs of transgender patients related to body image and the role of the body in traditionally structured ED treatment. Some clinicians were seen as blaming the ED entirely on the patients wanting to affirm their gender identity [3, 13, 14] and others accused patients of coming out as a means of attention seeking [14]. Most disturbingly, some clinicians suggested that symptoms of a transgender patient’s ED may lessen if the patient accepted the gender they were assigned at birth [3, 13].

Gender minority patients have clearly identified where clinicians may fail them throughout their ED treatment. The current analysis explores provider awareness of facilitators of and barriers to inclusive care for gender minority patients with EDs. Provider perspectives on these facilitators and barriers lend further insight into effective means to better address the unique health care needs of gender minority patients in the ED care setting. Through individual interviews with mental health clinicians caring for patients with EDs, we sought to learn more about provider perceptions of the barriers and facilitators to successful ED treatment for transgender and gender diverse patients and to elicit provider perspectives of the experiences of these vulnerable patients.

### Conceptual model

We evaluated provider perceptions and perspectives using deductive analysis guided by the Social-Ecological Model [15]. This model is built on the premise that health outcomes and healthcare experiences are the result of mutual interactions between individuals, groups, and their immediate and extended social environments. This model is particularly helpful in allowing those who seek to develop effective policies and health promotion measures and provide a richer understanding of the patient-provider interactions, clinic settings, the regulatory and policy environment, and broader social structural factors they seek to change [15].

## Methods

We conducted a series of individual semi-structured interviews with 19 clinicians in the United States, specializing in eating disorder treatment. Interviews addressed a range of topics related to their familiarity with transgender and gender diverse populations, perceptions of facilitators, and barriers to care, and opportunities for intervention and improvement of the delivery of ED care to transgender and gender diverse populations. The current analysis focuses on provider perceptions of gender minority patients’ experiences and challenges with eating disorder treatment.

### Setting and participants

We contacted potential participants from all US states and territories using email solicitation, social media messaging, and snowball sampling. We sought to recruit professionals with experience in a range of ED treatment settings and approaches to care and to capture a diverse sample. Eligible clinicians were older than 18 years, were fluent in English, had to have access to a phone or Zoom, were licensed to provide mental health treatment, and had at least one year of experience at their current role. Providers did not have to have experience working with gender minority clients to participate.

### Procedures

We identified mental health clinicians who specialized in ED treatment from the *Psychology Today* website (*Psychology Today*). All listed with a link to their website or an email address were contacted via email until sufficient representation was achieved for race, gender, and age. Additionally, clinicians were recruited via social media (Instagram) and personal academic networks (an ED interest email newsletter). Recruitment lasted 2 months. Potential participants who expressed interest in response to the recruitment messages sent via email or social media direct messaging were asked to complete a screening survey. Those who met our inclusion criteria were invited to participate and were sent the link to a background/demographic questionnaire. To improve the diversity of the pool of participants, we asked participants to identify potential respondents from their own networks who represented geographic and philosophic approaches to eating disorder care different from their own.

Web surveys and questionnaire responses for demographic and provider attitude and education data were collected and managed using REDCap electronic data capture tools [16, 17] hosted at the University of Massachusetts Chan Medical School. REDCap (Research Electronic Data Capture) is a secure, web-based software platform designed to support data capture for research studies. Participants provided consent electronically and confirmed being 18 years of age or older before completing the study background and demographics questionnaire. Questionnaires were completed before interviews; however, participants were told during the consent process that their survey responses would not be reviewed until after the interview when data analysis began. To ensure the anonymity of participants, each was assigned a participant ID. Participant names and email addresses with a matching participant ID were stored separately from participant data. All data were maintained on a secure research drive at the University of Massachusetts Chan Medical School.

Participants each met once with the interviewer (KF), via Zoom. KF is a cisgender female doctoral candidate with qualitative research experience. Semi-structured interviews were conducted using an interview protocol based on findings from Duffy et al. (2016) and Hartman-Munick et al. (2021) (See Additional file 1: Appendix A). Interviews were conducted in English and lasted 45–60 min. Elements of consent were verbally reviewed with participants prior to the start of each interview using an IRB approved Fact Sheet. Zoom audio recordings were downloaded onto secure University of Massachusetts Chan Medical School laptops connected to a secure verified network and transferred to a secure network drive for storage. Zoom video recordings and chat records were permanently deleted.

### Data analysis

Interviews were transcribed from Zoom audio files using Otter.ai. Transcripts were cleaned, deidentified, and verified by KF and EM. Final transcripts were independently coded by two researchers (KF and EM), who both identify as cisgender females. Coders met weekly to review coding discrepancies and new themes as they were identified. We used NVivo [18] for coding and employed inductive thematic analysis [19] to identify themes. Interrater reliability was calculated and found 94.3% agreement between coders.

## Results

### Characteristics of participant

Table 1 shows the characteristics of the 19 study participants. Participants were predominantly from the South, but geographic diversity was obtained, as was diversity age, race/ethnicity, gender identify, and practice setting.

**Table 1:** Participant characteristics of US-based mental health providers providing treatment for eating disorders (n = 19)

	Number of participants**
*Transgender identifying*	
Transgender	1
Cisgender	17
*Gender identity*	
Non-binary/non-conforming/gender fluid	1
Male	3
Female	14
*Racial identity*	
Asian/Asian American/Pacific Islander	1
Black/African American/African	2
White/Caucasian	13
Multiracial	2
*Ethnic identity*	
Non-hispanic or non-latinx	15
Hispanic or latinx	1
*Age group (years)*	
18–30	6
31–50	11
51 and older	2
*Clinic/practice type*	
Private practice	4
Outpatient treatment program	5
Community health clinic	1
Hospital-based clinic	2
Academic clinic or practice	1
College counselling center	2
Private clinic	1
Inpatient/residential treatment program	1
Other: peer led treatment program	1
Geographic region*	
West	3
Midwest	1
South	12
Northeast	3

*Regions, according to the US Census Bureau [31]

**May not total to 19 as some participants preferred not to answer some questions

### Gender identity and ED risk

Many clinicians discussed the increased risk for developing an ED among gender minority individuals (particularly youth) relative to cisgender (n = 6). Additionally, most clinicians noted that those undergoing puberty may use disordered eating behaviors to mitigate the impact of puberty on their bodies and reduce dysphoria (n = 10). Use of disordered eating for this purpose was perceived among clinicians as a risk factor for more moderate to severe ED among gender minority youth (n = 3). Higher risk was also attributed to gender dysphoria, limited accessibility of gender affirming care in the US, experiences of trauma (n = 11), physical violence (n = 5), and stigmatization (n = 8).

### Factors affecting access to care

Regarding barriers to ED treatment and patients’ experience within US mental health treatment settings, clinicians cited the following factors: stigmatization, family, financial factors, gendered clinics, scarcity of gender competent care, and religious communities. Quotes supporting these findings can be found in Table 2.

**Table 2:** Supporting provider comments on barriers to accessing care among gender minority patients seeking and receiving eating disorder treatment

*Stigmatization*
"I mean, family, lack of support, abandonment, rejection… that's a huge barrier, because how can someone get the help they need if their family is not helping them when they have a health rejecting illness?" (P01)
"I think, just like, past experiences with past providers are like a huge barrier where patients have been like, 'oh, I went to this one person, and this bad thing happened. So, why would I ever put myself in that situation again’, which is totally valid." (P18)
*Family support*
“…I've had a lot of people say, it wasn't until college, that they could even consider having access [to therapy], because the family didn't want the shameful secret, or that their child was not masculine enough or not feminine enough from their perspective, or, you know, the family didn't allow access to therapy with someone who would be gender affirming, and that was a bad thing, because it would only encourage this like delusion.” (P15)
“…the family education piece…specifically with this population, is to make sure that the parents are educated on not only good recovery skills, but also, why it's made more difficult or challenging in a situation where there are some identity things going on…so much of an eating disorder is about identity…we have to approach it as a whole person…we also have to make sure that your child feels comfortable being who they are in their skin…I think that helps parents have a little bit more buy in to the process.’” (P29)
*Financial factors*
"…a lot of the times the parent’s kind of cut them off, especially as they start to transition, they have a lack of support from various areas, and that includes financial. Eating disorder treatment is really expensive…some of our transgender clients might have to discharge early due to financial reasons and that's very sad." (P03)
“We've had clients in program who have been seeking gender reassignment surgery and for whatever reasons, financial or otherwise, maybe they couldn't afford it, and it could be like, ‘I have to choose between eating disorder treatment, or looking the way that I feel is appropriate. And I think that people are going to kill me in my community, because they won't accept me the way I am. So, it's like my eating disorder will kill me, or my community will kill me. So if I want to fit in, I have to get top surgery, but I can't afford it and eat at the same time.’ So sometimes people are having to make some pretty difficult decisions.” (P05)
"There was one incident with insurance, where there was no option for trans or gender expansive expressions. It was male, female, that was it. Because on their birth certificate, it said they’re a female, the insurance company wanted a female name. The female name on their bursary gave coverage to this individual for any kind of treatment. This patient was very flexible, very understanding, but I could see that being a barrier for a lot of folks…really invalidating, triggering, discouraging, and just like screw it, it's not worth it." (P11)
*Gendered clinics—cis female only*
“I've only worked in facilities that were female only, but some of our sister facilities…were coed. So we would have clients who would come to the place and say, ‘I don't feel comfortable, X, Y and Z.’ So we say, ‘Okay, well, we can transition you to one of our sister facilities that's in Florida or California,’ but they don’t have resources to get to Florida or California. So, it kind of just puts them in a crunch.” (P21)
*Scarcity of gender competent care*
"One of the states I've worked in is not a very liberal state, and can be kind of dangerous, sending certain clients to certain providers…the state of Texas, living there and working with several trans clients, I wasn't scared, but I just felt for them and wanted to make sure that I was sending them to doctors that were educated and appropriate with this population." (P07)
"The state of Kentucky is not a super welcoming place for anyone who isn't a heterosexual cis white man…I will say we're located in the biggest city in Kentucky so, I do think because it's a metropolitan area, it's more accepting…tolerant. I don't know that folks are like, necessarily feeling actively positive about the trans population but I think they're certainly more open than in some of the rural, small towns. And like if you don't want…somebody to see you walking into the clinic, even if they're not coming here…it might be hard to have that privacy" (P11)
*Religious communities*
"It's a very conservative and a very religious community that is not LGBTQ affirming, that is not, in general, supportive of gender diverse individuals…So I think the barriers are both practical and financial…a huge way that people access services in this community is through like a religious or ecclesiastical provider. They provide the referral and sometimes payment for services. It gets very, very thorny there because the person that is basically in a position of power, that religious figure, to pay for those services and may only refer to people who are not necessarily gender affirming." (P27)
"So my practice is in a predominantly conservative, religious community…there are unique challenges to that for our populations that we're serving, because a lot of them are coming into our clinic, with beliefs about themselves that are quite negative or distressing to them personally…so, if they're living a life that they feel is outside of that, it's really eye opening [for them] to sit with someone and to have that person say, 'I think that you are still someone that can live your values and live your life, authentically and that's not such a black and white issue.'." (P29)

#### Stigmatization

Most felt that gender minority patients’ fear of stigmatization or trauma triggered from prior experiences can prevent them from seeking treatment (n = 10). Clinicians recognized themselves, patient peers, as well as the family of patient peers, and patients’ own family members as having roles in shaming, silencing, or rejecting gender minority individuals. A few clinicians acknowledged their historical role as gatekeepers in accessing gender affirming care (n = 3), which they felt has contributed to gender minority patients’ distrust when seeking out psychological services, including ED treatment.

#### Family support

Clinicians perceived a lack of family support as negative in its impact on gender minority children seeking ED treatment (n = 19). According to several clinicians, it was not uncommon for unsupportive parents to limit their children to treatment facilities and clinicians who adhered to parental values and beliefs around gender identity (n = 10), particularly in religious communities (n = 3). Some clinicians observed that patients with unsupportive family members delayed seeking care or coming out until they were living independently as adults, to avoid familial and community conflict as youth (n = 3). They speculated that, as a result, gender related distress and ED symptomology were likely prolonged. Many families, however, were described as supportive, which allowed patients to easily access care. According to clinicians, many parents who, in one way or another, receive education around gender identity and eating disorders may be less likely to hinder their child’s ability to continue in or pursue care with affirming clinicians (n = 6).

#### Financial factors

It was noted that many clinicians and clinics (generally) treating EDs do not accept insurance. Cost of care was viewed as a great impediment for patients who may need to choose between costly gender affirming medical intervention or ED treatment when their safety and wellbeing may require both (n = 9). Several observed that most patients could rarely afford both types of care (n = 7).

Clinicians associated lack of financial support for ED treatment with lack of gender affirmation from family (n = 5). A few participants reflected on instances where young gender minority individuals were kicked out of their homes, having to support themselves with low-income jobs often without health insurance (n = 3). A concern was that those with insurance may find that ED care is not covered due to incongruence between gender assigned at birth and their current gender identity (n = 2).

#### Gendered clinics—cis female only

Clinicians highlighted that female-only residential treatment facilities, may be unappealing to gender minority patients even if they are welcomed by staff (n = 3). Patients may try to seek care elsewhere, but other mixed gender facilities may be less financially feasible to access due to 1) distance from the patient (n = 4) or 2) because facilities have staff who are discriminatory or not gender competent (n = 7).

#### Scarcity of gender competent care

Several clinicians noted their gender minority patients struggled to identify safe clinicians located outside of their city or state (i.e., mental health clinicians and other healthcare specialists) (n = 7). This struggle was thought to be exacerbated by a scarcity of gender-competent ED treatment professionals (n = 5). Accessing affirming or competent ED care was generally felt to be more difficult for those living (1) where care in general is scarce (patients may need to travel to access it) (n = 4) and (2) in states with more conservative political leanings (n = 5) which was believed to be a barrier as patients may be unsure of whether a provider’s political ideology facilitates discrimination towards gender minority individuals.

#### Religious communities

A few clinicians were familiar with the struggles of gender minority individuals living in communities with large religious populations. Clinicians who disclosed that they work or have worked in these communities (n = 4) explained that gender minority clients seeking care were often hindered by spiritual leaders and religious family members (n = 4), more so if care affirmed gender identities that were not felt to be compliant with religious doctrine (n = 2). Two of the four clinicians conveyed that it was not uncommon for gender minority patients to avoid care or struggle throughout care because they feel their identity conflicted with their religious values. Some participants felt that clinicians in religious communities are often not prepared to help patients grapple with seemingly conflicting identity and spirituality (n = 2).

### Factors affecting care while in treatment

Clinicians cited the following factors affecting gender minority patients’ care while in ED treatment: discrimination and microaggressions, provider lived experience and education, other patients and parents, systems of higher education, family-centered care, gender-centered care, and traditional therapeutic techniques. Quotes supporting these findings can be found in Table 3.

**Table 3:** Supporting provider comments on barriers to successful care experiences among gender minority patients seeking and receiving eating disorder treatment

*Discrimination and microaggressions*
“…the boss that I left from, they were also an older male, who was very open about sexuality but when it came to gender and pronouns…they refused, because it wasn't grammatically correct. Like, we helped a lot of patients come out to their families and created a safe space but when it came to that (gender), there's no tolerance.” (P16)
“…we're like, okay, this person needs to go to this specialist…they need to have their gastro system looked at more carefully…the doctor on our team was like, ‘I mean, we can send them there and that's the only place we can send them but it's gonna be rough’, and then you try to help the client through it…prepare them what to say, how to navigate, what to expect. Like literally like you have to send them into an unsafe space…in Arkansas we don't have a lot of choices…there's only one or two doctors in all of the state…so when they need a specialist, it's really risky for them as far as their identities.” (P10)
“…there's a piece of legislation in this state it's called the Conscience Clause. So, essentially, it gives people (providers) a pass (on religious or conscience grounds)…we've had clients that said, ‘this person seemed okay or they were the only one that took my insurance, I had appointments,’ and then the person is telling him, ‘I need to refer you out because I don't have enough experience to treat you.’ They're back in the same place of, ‘I've been vulnerable (disclosed gender ID) and that person told me, I can't help you.’” (P17)
*Provider lived experience and education*
"I think a much more fundamental issue is the lack of diversity within our fields…especially for the kind of treatment that I do in inpatient residential settings, where you're really living with your carers (sic)… you're not seeing somebody once a week for an hour. I think especially in that context, for the people around you not to be reflective of your experience, is a huge problem. It doesn't matter how well educated they are, it doesn't matter how well intentioned they might be, it doesn't matter how many seminars they might have taken, there's a fundamental difference in having some level of lived experience of the nuances that come along with whatever you might be talking about and just learning about something in an academic way." (P25)
*Other patients and parents*
“I think, unfortunately, all levels of their care can be impacted, especially if they're male…or if they identify as male. It also makes it tricky, because I work with other clinicians at the residential part of our company and housing is really difficult with these particular patients because parents of [other] adolescents do not feel comfortable with a patient in their say, daughters, room who's heterosexual for all we know. They are 12, but you know, who knows? But then there is a 13-year-old that is transitioning, they find that out, then they cause a ruckus!” (P16)
“My patient that I worked with for a while talked very specifically about someone who had this ‘Back the Blue’ poster in her picture [in telehealth video group therapy] and me saying to him, 'so you're probably very fair about what those assumptions are, but we don't 100% know. And just because she has someone in her life, who is a police officer, doesn't mean that she doesn't accept you as you are. So, I get it, and I get why you're wary and let's also try to be fair, and if she says anything, let me know, because we're going to squash that very quickly and it's not okay.'…it's challenging.” (P02)
*Institutions of higher education*
"So our board…they hold a lot of power. The politics of my state have a big impact…we wanted to put out a statement, during some of the events that have been happening over the last couple of years, making sure that our students knew that we were welcoming of all people, specifically, our gender minorities…and we were not allowed…it's really run by the views of the board [of the university] and what messaging they want us to put out. So, we've got people that push and advocate but when it comes down to even little things, like changing the paperwork that our clients see when they come in so it's not, 'identify yourself as male or female'…it took a lot of time for us to get that approved.” (P10)
*Family-centered care*
“…a different nonbinary client…this person is only 11 years old…I kind of helped them go through that process of like telling their family and telling their doctors and everything like that. But that was incredibly difficult. And mom just emailed me and is still using the incorrect pronouns…apparently, they're using a completely different name now, and mom is still using the old one…and I think, working with families in this population…like this person is 11…I find really, really challenging because it's like, I don't want to step on these parents toes in any way, but I also want to advocate for my client.” (P07)
“some of the values and attitudes of family members…parents and family just kind of emotionally cut them off…a sense of personal rejection…so you've got that compounded on their sense of identity confusion that they've gone through on that, and trying, again, to be accepted by others…all the negative self-talk that goes on with an eating disorder, it just seems like it's compounded or exponentially higher, with an individual who is transgender…there have been other family situations or support systems where their partners have been very supportive…you need a support system in eating disorder recovery.” (P09)
*Gender-centered care*
“…a lot of treatment facilities are female only. That's going to be a big, big issue…I've worked in a clinic where we accepted a transgender male….and this client who was transgender, transitioning from a female to a male, he kind of felt, you know, like an outcast, an outlier because even though he had female body parts he identified as a male and a lot of the things that we were teaching and the discussions about the body that we were having you relate to more so as a woman, so that was kind of difficult for him.” (P21)
"So, I think even for cis males who have eating disorders, I know that most, if not many of them, would probably feel very uncomfortable in most treatment centers, just because of how gendered most treatment and the content of treatment tends to be…anybody who's not at least female identifying would probably have a similar experience. Layer on top of that the relative lack of comfortableness in most healthcare settings, for somebody who's not cisgender and who probably is not straight either. The lack of sort of general competency in those realms…lack of welcomeness in those kinds of settings…I think that also then magnifies that disconnect for people coming into treatment…I don't think we're doing a great job." (P25)
“There was one point where I was filling in over there just because they moved us around to where people were needed the most. We had a client that was trans that identified as female, and they had them on the male unit, which even at that point, being as ignorant as I was compared to now, I would say, to me, that didn't seem quite right. And I asked about it and essentially, they were saying, this is the best fit [for the patient] based on how it was going. There were a lot of issues with that.” (P05)
*Traditional therapeutic techniques*
"You have providers trying to press acceptance of one's body shape and weight, or managing eating style without understanding gender dysphoria, and one's already complicated relationship with their body could actually exacerbate gender dysphoria. So, by having tried some of the typical interventions, like looking at a mirror and telling yourself positive things isn't going to work…it would be like the antithesis of your identity to have to be forced to accept certain parts of your body." (P15)
“…exposure, meal outings and going out in the world and eating…going certain places or clothing shopping…they might not feel fully comfortable. You know, getting looks from people or getting questions and, ‘why are you in the women's section?’…’why are women in the men’s section?’… things that are even as seemingly simple as picking a dressing room when you're out in public trying to do those shopping exposures, I can see all of that being not impossible, but just an additional barrier for us to have to consider when trying these different therapeutic interventions.” (P11)

#### Discrimination and microaggressions

Clinicians considered lack of acceptance and validation or microaggressions from other clinicians, peers in treatment, and family as barriers to successful treatment and sources of trauma for gender minority patients. Clinicians broadly associated discrimination from clinicians with being older (n = 4), having restrictive religious or political beliefs (n = 10), limited knowledge around gender identity (n = 5), and stigmas around gender identity (n = 7). Some interview participants spent time preparing gender minority patients to cope with other clinicians, usually specialists outside of their practice, whose beliefs were unknown or were known to be discriminatory but were the only option for these patients (n = 2).

#### Provider lived experience and education

Many clinicians perceived the mental health community broadly as well as those providing ED treatment to be poorly trained to work closely with gender minority patients (n = 17). Some clinicians discussed how patients are frequently burdened with educating clinicians about gender identity generally and as it applies to their eating disorder (n = 11). A handful of clinicians recognized that while patients are excellent first-hand resources, they should not have to take on the task of bringing clinicians up to speed on how to provide gender informed treatment (n = 5). Some clinicians discussed the benefits of programs that had staff who reflected the gender, racial, and ethnic identities of patients; stating that programs with diverse staff were more effective in their ability to provide a safe, informed, and relatable space for patients with similar identities (n = 7).

#### Other patients and parents

Among clinicians who offered it, group therapy was often deemed an inclusive space for gender minority patients, particularly when practices/clinics had established rules for interpersonal respect in that setting (n = 4). Use of telehealth platforms for group therapy raised some concerns about potential harm, such as inadvertently exposing patients’ personal beliefs (i.e., posters, signs, flags*—*in video background) or exposing identities of group members to others in a household or workplace, which otherwise may have never surfaced in an in-person clinic session (n = 2). Clinicians felt that instances such as these had reduced comfort for gender minority patients. Additionally, numerous clinicians noted that it was not uncommon for a roommate’s parents, unsatisfied with their child’s roommate’s gender identity, to either remove their child from treatment at that clinic or have them relocated to another room (n = 5). It was also mentioned that some parents of other patients had made bigoted comments about gender minority patients (n = 3).

#### Institutions of higher education

One provider felt that despite the counselling center at their religious university making efforts to provide safe and inclusive care, the broader university culture often counteracted much of that work. Of clinicians who acknowledged having experience working in college counseling centers (n = 5), three reported feeling that they were often a patient’s only source of affirmation, and that patients with limited support outside of the clinical setting may struggle to excel in their recovery. Additionally, two noted that gender minority patients may require several sessions before feeling safe and ready to be transparent with their provider. This may limit progress patients and clinicians can make with ED recovery, as students are typically only on campus for 2–4 years and may only be allotted a fixed number of sessions per year.

Additionally, counseling center clinicians’ efforts to make materials more inclusive were sometimes slowed by institutional procedures, policy or culture and influenced by donors, politicians, or personal beliefs of institutional leadership (n = 4). Some described pushbacks from leadership when seeking to develop and promote inclusive messaging and programming, which they felt was necessary for their clinics to promote based on the political actions of their states towards transgender individuals, in recent years (n = 2). Another clinician indicated that in addition to their institution’s religious teachings preventing them from having an LGBTQ specific group therapy program for years, once the group did receive approval, they could not title the group as being an LGBTQ support group due to the discriminatory culture on campus.

#### Family-centered care

Clinicians had a range of experiences with families of their gender minority patients; some with many supportive parental encounters and others reporting few to no encounters with supportive family. The role of the family is often central to ED treatment, primarily for youth. This was mentioned by most providers (n = 16). The majority of clinicians described family-centered care as most effective when parents affirmed their child’s identity (n = 15). Families who made efforts to self-educate or who were open to being educated by clinicians were viewed as great sources of support in their child’s ED recovery (n = 6). Conversely, clinicians recalled instances of unsupportive parents extracting their children from ED treatment if their child’s identity was being affirmed by clinicians (n = 5). Unsupportive parents reportedly exhibited counterproductive behaviors while their children were receiving care, having persisted in efforts to invalidate their child’s identity by using their old name (deadnaming) or misgendering them (n = 7). Several clinicians feared accidentally outing patients, while trying to affirm their identity and tiptoeing around difficult or toxic family members (n = 7).

#### Gender-centered care

In some female-only facilities, where transmasculine patients (female transitioning/ed to male) were receiving care, clinicians expressed concern for: (1) patients not feeling included in therapeutic programming due to their gender identity (n = 4), (2) therapeutic focus on the cisgender female body (n = 7), (3) provider unfamiliarity with working with patients identifying as male or non-binary (n = 6), and (4) a lack of neutral facilities (i.e. restrooms)*—*all of which may cause distress for gender minority patients in recovery (n = 3). Clinicians looked negatively upon instances where gender minority patients were placed in care wings for the gender they were assigned at birth, rather than their current gender identity (n = 4).

#### Traditional therapeutic techniques

A lack of knowledge around therapies that accommodate ED patients with gender dysphoria was discussed by many clinicians (n = 12). Most clinicians felt available ED treatments were not designed with transgender or gender diverse bodies in mind (n = 8); many felt unprepared to navigate providing non-triggering treatments to gender minority patients, especially to those undergoing medical transition (n = 9). Consequently, patients communicated feelings of distress to clinicians about traditional approaches to care such as observing their body in a mirror, engaging in body-positive and body acceptance talk, and exposure therapies such as residential facility field trips to practice body acceptance in retail clothing stores. Clinicians reported instances of gender minority patients feeling triggered around body-centric activities, especially in gendered settings such as fitting rooms or clinic restrooms (n = 3). Several clinicians noted that their clinic or other clinics had made changes to their facilities to provide focused programming for gender minority individuals (i.e., Intensive Outpatient Program, Partial Hospitalization Program) (n = 7). According to these clinicians, patient response was overwhelmingly positive, and programming was well utilized by members of these populations.

## Discussion

Provider perceptions in the current study were in line with findings from previous studies of patient perspectives, focused on provider and systemic influences, which have hindered patients from productive and safe eating disorder treatment. Our analysis generated two overarching themes: (1) barriers to accessing care/finding provider and (2) barriers to receiving therapeutic and safe care. Many barriers to accessing care and barriers to receipt of effective and safe care were similar but had differing impacts.

In our study the role of family in eating disorder treatment and in the lives of gender minority patients was prominent. Clinicians in the current study noted the need to consider how the attitudes of their patient’s families towards their gender identity can impact success in treatment; a barrier not discussed in patient studies. The role of family in eating disorder treatment may cause unease, particularly among those who are not yet out to their families or clinicians. Many gender minority patients don’t come out at all throughout treatment to avoid mistreatment, discrimination, or other burdens imposed on them by clinicians while they are seeking help for their eating disorder [3]. Clinicians in our study noted that patients may wait to come out until they are living independently for similar reasons. Previous studies with patient participants have largely explored care experiences in those older than 18 years [3, 13, 14]; our study allowed clinicians to reflect on the experiences of patients of all ages, including the experiences of younger patients, ranging from middle school to college, who are more impacted by the role of family, and adult patients who are more independent and less impacted. Research to better understand how family centered therapeutic styles may serve as barriers to care for gender minority youth is warranted.

Clinicians in our study honed in on unique roles that patients’ social contexts and communities have on their ability to access and receive quality eating disorder care. The use of religion to justify denial of care disproportionately impacted gender minority individuals—under Conscience Clauses. The language of Conscience Clauses affords clinicians the right to refuse to provide care to transgender individuals if they feel that (1) they would need to discuss gender affirming medical intervention in any way or (2) if a condition is so much as tangentially associated with the patient’s gender identity [20]. A 2016 Center for American Progress survey found that 29% of transgender individuals were refused the ability to even see a health care provider, because of provider attitudes towards gender minority individuals [21]. Twelve percent managed to see a health care provider but the provider, because of their religious beliefs, refused to provide affirming care to the patient.

Institutions of higher learning can have direct roles in selecting what care is available to their students who use on-campus health services. According to clinicians, influence from upper leadership on campuses has resulted in an inability to use inclusive messaging to welcome gender minority patients or to provide certain types of healthcare for these individuals. Barriers to counseling center treatment were a novel finding in this study and should raise cause for concern, as gender minority individuals entering college counseling centers for care are more likely to have more severe mental health concerns than their cisgender peers [22]. Limiting or denying access to safe, affirming eating disorder care can delay a patient from entering care for their eating disorder or other treatment for other mental health conditions. Gender minority college students are 4–6 times as likely as cisgender college students to be diagnosed with an eating disorder [2]. Additionally, eating disorders increase risk for suicide and suicidal ideation, especially among gender minority individuals [23]. Risk for suicide or suicidal ideation can be exacerbated if patients are unable to access affirming care or are subjected to transphobia [24, 25]. For these reasons, the barriers that clinicians identified in these care settings serve as cause for concern. Improving access to affirming care and accommodating the needs of gender minority students on college campuses is imperative.

Clinicians in our study who worked on religious affiliated campuses and within religious communities mentioned methods of accessing care through ecclesiastical leaders as a means of getting eating disorder treatment. Despite access to care through religious pathways, transgender patients were hesitant to do so as the gender they identify as or avoided care until later in life. The religious affiliations for campus clinicians in this study did not support receipt of gender affirming care and clinicians noted that this had detrimental effects on their gender minority patients. Religiosity has been shown to be associated with negative attitudes towards gender minority individuals [26, 27]. Religiosity of a community in which a gender minority individual lives or practices their faith has been identified as a barrier for them in seeking to access health care generally [28]. However, there is a paucity of research exploring how access to care through religious pathways has impacted ED outcomes and experiences for gender minority individuals.

Study participants were particularly aware of the ways in which discrimination manifested and impacted patients. In prior studies, avoidance of treatment due to misgendering, or continued use of an old name (i.e., deadnaming), and/or clinicians using incorrect pronouns were noted as major barriers that prevented gender minority patients from receiving care for their eating disorder [29]. Twenty three percent of gender minority patients receiving any type of healthcare have reported being misgendered or deadnamed by a provider [21]. Our participants confirmed that it was not only difficult for patients to find affirming clinicians, but it was difficult for clinicians themselves to identify safe clinicians for referrals. Gender minority patients have expressed frustration with the uncertainty of safety or acceptance when seeking out a provider for eating disorder treatment—preferring that clinicians clearly identify themselves as allies of transgender and non-binary patients [13]. Although many clinicians may claim to be accepting, both patients and clinicians have found that clinicians who self-identify as allies are not always living up to that expectation [13, 14]. Clinicians and patients who identify as transgender or non-binary have expressed that those who have lived experience may be best suited to work with patients with similar gender identities; these shared experiences likely serve as a source of comfort to patients who may not wish to be so transparent with cisgender clinicians [13, 14]. Many patients have expressed greater feelings of comfort working with gender minority clinicians [14]. Patients have attributed these feelings of comfort to gender minority clinicians having a higher likelihood of affirming a patient and greater gender-competency by way of personal experience [14].

In treatment spaces, both clinicians and patients called attention to the ineffectiveness of traditional ED treatment approaches and the detrimental effects they have on gender dysphoria for many gender minority patients. Patients from prior research felt like the use of body positive approaches, such as radical body acceptance, make them feel like they are “pushing themselves to be in their body” [13]. Clinicians in our study elaborated further, noting that their gender minority ED patients have a difficult time with exposure therapies and discussions around anatomies of cisgendered bodies. Gender minority patients have expressed discomfort with gendered care settings that exclude the body experiences of transgender and non-binary individuals [3, 13, 14]. The ability to use names and pronouns to involve gender in ED treatment by clinicians is necessary, but not sufficient. Patients report that clinicians blame their ED for causing gender dysphoria and others blame the dysphoria for causing their ED [14]. Most clinicians in this study recognized that dysphoria is not always the cause of ED for every gender minority patient; their ED may result from trauma, poor body image, or anxiety. Patients have asked for care needs to be tailored to the individual such that clinicians inquire about gender identity and how it is affecting the patient individually, not as a member of a homogenous group [14].

### Strengths and limitations

Complementing research including patients, this study focused on the views of clinicians, who may have a fuller picture of care experiences across multiple patients of varying ages, races and ethnicities, experiences in medical and social transition, support systems, and financial circumstances. Individual interviews allowed for extensive discussion of provider perspectives and experiences. Their views elaborate on the narratives of patient perspectives in other studies; specifically, views on provider comfort and ability to treat gender minority individuals with body-centric mental health conditions, as well as the dominant disparities in this field of care. This study provided responses from nineteen clinicians of differing ages, race/ethnicities, clinical experiences, clinical settings, and geographic locations. Our sample was representative of provider demographics in the United States [30].

Validity in qualitative research can be impacted by reactivity, researcher bias, and selection bias. We sought to include clinicians from a wide range of belief systems and attitudes towards gender minority individuals. Yet, participants may become aware of researcher preconceptions on the subject matter and feel compelled to alter responses. We made efforts to reduce participant reactivity prior to the start of each interview by having the interviewer explicitly convey that all (1) perspectives, (2) beliefs, (3) maintained knowledge and (4) lack of knowledge on the subject matter were critical and equally important to researchers in this study.

The lead author (K.F.), who served as the interviewer and as a coder, had extensive prior knowledge of barriers and facilitators to care for gender minority individuals in U.S. mental health and primary care settings. Prior content area knowledge may have biased how codes were constructed and populated. To reduce the impact of researcher bias, we employed a second coder (E.M.) with limited prior knowledge of the subject matter and population. Both K.F. and E.M. identify as cisgender females, which may have limited the scope through which data could be understood, as neither had lived experience as a gender minority individual. Both coders made concerted efforts to question whether their own beliefs and assumptions were leading them to interpret findings a particular way.

Despite these strengths, study findings must be considered with limitations in mind. We recognize that we may have missed perspectives of clinicians with radically differing attitudes toward gender minority patients given the voluntary nature of participation. Future studies should aim to focus on the perspectives and views of (1) clinicians with complete lack of knowledge/inability to make assumptions about this population’s care experiences, (2) clinicians from cultural communities that have strong beliefs about traditional gender roles, (3) clinicians who are explicitly bigoted, and (4) clinicians who feel that gender minority individuals face no difficulties in life or ED care.

### Implications

Clinicians demonstrated awareness of barriers to care for gender minority ED patients but expressed great uncertainty about methods of appropriate care delivery in this population. They expressed concern regarding lack of sufficient education about effective ED care for this population. Our findings highlight a need for understanding the provider level limitations in training and potential for system change in the care space that would address needs identified by gender minority patients and their advocates.

## Conclusion

Clinicians in our study were aware of a plethora of barriers to care facing gender minority patients that impair their ability to receive effective treatment for eating disorders. Improvements to eating disorder treatment spaces, provider knowledge, and treatment approaches to better serve gender minority patients are warranted. Future research should expand upon extant studies that have identified weaknesses in the education of mental health clinicians, as well as clinicians’ ability and willingness to practice gender affirming and inclusive care.

## Supplementary Information

**Additional file 1: Appendix A.** Interview protocol.

## Data Availability

The datasets generated and analyzed during the current study are not publicly available because we did not obtain explicit consent to share these data beyond the study team. As such, we are unable to share the qualitative data collected as part of this study.
