# Supplementary material for: Provider perceptions of barriers and facilitators to care in eating disorder treatment for transgender and gender diverse patients: a qualitative study
Source: J Eat Disord. 2023 Mar 8;11:36. doi: 10.1186/s40337-023-00760-9 (PMC9993680; doi:10.1186/s40337-023-00760-9)
Supplement: Supplementary file 1 — Additional file 1: Appendix A. Interview protocol. [file 40337_2023_760_MOESM1_ESM.docx]

**Appendix**

**Interview protocol.**

| **Objective – To Investigate** | **Questions** |
| --- | --- |
| Personal experiences with transgender and gender diverse patients. | 1. During your time as a practitioner at your current practice or clinic, have you provided care to a transgender or gender diverse patient?    1. Were there challenges, ease, working with gender minority patients?    2. How did you adapt or personalize your approach to care? 2. If a patient presented to you with an eating disorder and gender dysphoria, how would you typically get started or proceed?    1. What types of questions would you ask the patient?    2. Would any questions you ask them differ from questions you would ask someone who is cisgender? Why or why not? 3. How confident are you in your skills necessary to effectively treat an eating disorder with a patient who is transgender or gender diverse?    1. Do you feel prepared to provide gender inclusive and/or affirming care?       1. In what ways do you feel prepared; in what ways do you not? |
| Coordinated gender affirming care, care networks, and clinic climate. | 1. Tell me about your comfort with engaging in coordinated gender affirming care with medical professionals outside of your clinic/practice… (i.e. would you feel comfortable writing a letter of support for gender affirming surgical intervention for a patient?)    1. If you have worked with patients who have come from other providers, have they said anything of note about their experiences with other providers related to their gender identity? Maybe 1 or 2 experiences or any generally common experiences? Good, neutral, or bad? 2. Do you feel comfortable referring a transgender or gender diverse patient to another provider if you cannot provide care they need?    1. What factors would lead you to refer a transgender or gender diverse patient with an eating disorder?    2. What factors would influence who you refer that patient to?    3. At this time do you know other providers, outside of your practice/clinic who provide gender inclusive care? Inside your clinic?    4. If you did not know of one, how would you go about identifying a provider for a gender minority patient? 3. Tell me a bit about the climate in your office or clinic surrounding the delivery of care to transgender and gender diverse patients.    1. What are some of the prevailing opinions among the staff about care needs of transgender and gender diverse patients?    2. What is the general familiarity of staff and other providers with gender affirming care? |
| Self-education | 1. Tell me a bit about your personal engagement with gender affirming care practices, literature, and other resources for self-education?    1. Which resources have you personally used?    2. Which resources have been useful, not that useful, pointless?    3. If you have never used any resources, where might you go to find them if you were hoping to learn something about gender identity? 2. Tell me about your education in college or university surrounding gender identity?    1. Were there classes specific to gender identity?    2. What topics were covered related to gender identity?    3. Did you feel like your education around gender in college/university prepared you for working with gender minority patients? In eating disorder treatment? |
| Perceptions of how patient gender identity impacts eating disorder treatment. | 1. From your experience and perspective, how might a patient’s gender identity impact their eating disorder care? Their mental health? 2. How might the care that transgender and gender diverse patients receive differ from or be similar to care provided to cisgender patients with eating disorders? |
| Perceptions of barriers to care and evaluation of how barriers may impact care. | 1. In the treatment of eating disorders, a support system can be valuable to a patient. How might you approach building a treatment plan that incorporates a support system for a transgender or gender diverse patient? 2. What barriers to accessing care might transgender or gender diverse patients experience when seeking eating disorder treatment?    1. I.e. they haven’t found a provider yet but are looking for one…what would impact ability to access care? 3. What barriers to appropriate care might transgender or gender diverse patients experience while actively receiving treatment for their eating disorder?    1. I.e. they are working with a provider, what might impact their care experience or desire to remain in care? 4. What has most impacted your perception of the needs of gender minority patients with EDs?    1. Most impacted your understanding of gender minority individuals generally speaking? 5. Aside from those which you may have already mentioned: what changes/improvements, if any, do you feel are needed to improve eating disorder treatment for gender minority patients? |
